# Complete Genome and Plasmid Sequences of Three Fluviibacter phosphoraccumulans Polyphosphate-Accumulating Bacterioplankton Strains Isolated from Surface River Water

**DOI:** 10.1128/MRA.01474-20

**Published:** 2021-03-04

**Authors:** Wataru Suda, Yusuke Ogata, Lena Takayasu, Chie Shindo, Keiji Watanabe

**Affiliations:** a RIKEN Center for Integrative Medical Science, Yokohama, Kanagawa, Japan; b Department of Human Ecology, School of International Health, Graduate School of Medicine, The University of Tokyo, Tokyo, Japan; c Center for Environmental Science in Saitama, Kazo, Saitama, Japan; Georgia Institute of Technology

## Abstract

Fluviibacter phosphoraccumulans is a polyphosphate-accumulating freshwater bacterioplankton which is detected mainly from riverine environments. The type strain, SHINM1, and two other strains, ICHIJ1 and ICHIAU1, were isolated from surface river water in Japan. Here, we report the complete genome and plasmid sequences of three *F. phosphoraccumulans* strains.

## Abstract (precis)

Fluviibacter phosphoraccumulans is a polyphosphate-accumulating freshwater bacterioplankton which is mainly detected from riverine environments. The type strain, SHINM1, and two other strains, ICHIJ1 and ICHIAU1, were isolated from surface river water in Japan. Here, we report the complete genome and plasmid sequences of three *F. phosphoraccumulans* strains.

## ANNOUNCEMENT

Fluviibacter phosphoraccumulans belongs to the family *Fluviibacteraceae* of the order *Rhodocyclales* of the phylum *Betaproteobacteria*. The taxonomic assignment was conducted by the combination of phenotypic (e.g., respiratory quinones, fatty acids, and polar lipids) and genotypic (e.g., 16S rRNA genes and genome phylogenies, average nucleotide identity [ANI], digital DNA-DNA hybridization [dDDH], Genome-to-Genome Distance Calculator [GGDC], and average amino acid identity [AAI]) characteristics (1). The *F. phosphoraccumulans* strain is positively stained with intracellular polyphosphate granules by Neisser and 4′,6-diamidino-2-phenylindole (DAPI) staining, and thus it is a polyphosphate-accumulating bacterium. A total of 204 strains of the genus *Fluviibacter* have been isolated from freshwater samples, which were collected mainly from surface river water and partly from surface lake water in Japan (2, 3).

Here, we report the complete genome and plasmid sequences of *F. phosphoraccumulans* strains SHINM1^T^ (JCM 32071^T^ = NCIMB 15105^T^), ICHIJ1 (JCM 33383), and ICHIAU1 (JCM 33382). Strains SHINM1^T^, ICHIJ1, and ICHIAU1 were isolated from surface river water samples in Japan (1). The river water samples were filtered through a disposable syringe equipped with a 0.7-μm particle retention glass fiber filter (Pradisc 25 GF/F disposable filter device; Whatman, Springfield Mill, UK). Filtrates were spread onto modified Reasoner’s 2A (MR2A) agar plates and incubated at 27°C for 3 days (4). A single bacterial colony was picked and inoculated into sterilized MR2A liquid medium (pH 7.2). This medium was incubated at 27°C for 2 days with reciprocal shaking (120 rpm). The pure strain cell suspension was stored in a sterilized aqueous glycerol solution (final concentration, 20% [wt/vol]) at −80°C. Each strain of *F. phosphoraccumulans* in glycerol stock was inoculated and cultivated in MR2A liquid medium, and the cells were harvested by centrifugation for genomic DNA extraction.

The genomic DNA of strains SHINM1^T^, ICHIJ1, and ICHIAU1 was extracted with enzymatic digestion as previously reported (5). Whole-genome sequencing of these strains was performed with MiSeq (Illumina, Inc., San Diego, CA, USA) and Sequel (Pacific Biosciences [PacBio], Inc., Menlo Park, CA, USA) platforms. The libraries of the MiSeq (2 × 300-bp paired-end) and Sequel platforms were prepared using the TruSeq DNA PCR-free kit (target length, 550 bp) and the SMRTbell v. 2.0 template preparation kit without DNA shearing, respectively. The MiSeq reads were trimmed and filtered with a >20 quality value using FASTX-toolkit v. 0.0.13 (http://hannonlab.cshl.edu/fastx_toolkit), and error correction of the sequel reads was performed using Canu (v. 1.8) (6) with additional options as previously described (7). Both sets of quality-passed reads were assembled using the hybrid assembler Unicycler (8), which contained a check of the generated genome circularization. The obtained genome sequences of the strains SHINM1^T^, ICHIJ1, and ICHIAU1 were annotated using DFAST (https://dfast.nig.ac.jp) (9). Default parameters were used with Unicycler and DFAST, and data from the obtained reads and generated genome sequences are described in Table 1.

**TABLE 1:** Information from the obtained reads and contigs

Characteristic	Data for strain:		
	SHINM1^T^	ICHIJ1	ICHIAU1
No. of quality-passed MiSeq paired reads	759,848	773,468	869,502
Total no. of bases of quality-passed MiSeq paired reads	453,222,422	461,573,550	516,845,415
Avg length of quality-passed MiSeq paired reads (bp)	298.2	298.4	297.2
No. of quality-passed Sequel reads	121,301	80,068	121,265
Total no. of bases of quality-passed Sequel reads	2,532,278,986	1,063,926,837	1,650,297,317
*N*_50_ of quality-passed Sequel reads (bp)	27,119	18,658	18,709
Total no. of contigs (chromosome, plasmid)	1, 1	1, 1	1, 1
BioProject accession no.	PRJDB6461	PRJDB9206	PRJDB9207
BioSample accession no.	SAMD00098160	SAMD00201023	SAMD00201024
Sequence Read Archive (SRA) accession no.	DRX145680, DRX196080, DRX196081	DRX195725, DRX195726	DRX195727, DRX195728
Genome size of chromosome (bp)	2,295,374	2,431,578	2,392,860
GC content of chromosome (%)	54.3	54.2	54.2
GenBank/ENA/DDBJ accession no. of chromosome	AP019011	AP022347	AP022345
Genome size of plasmid (bp)	9,965	16,356	16,356
GC content of plasmid (%)	51.0	54.6	54.6
GenBank/ENA/DDBJ accession no. of plasmid	LC523991	AP022348	AP022346

In accordance with annotation results, the genomes of strains SHINM1^T^, ICHIJ1, and ICHIAU1 had two genes for polyphosphate kinases (*ppk1* and *ppk2*), which were related to the intracellular accumulation of polyphosphate. On the other hand, these three strains lacked ATP-dependent glucokinase, which was related to the phosphorylation of glucose to glucose-6-phosphate and catalyzed the first step in glycolysis.

The average nucleotide identity by orthology (OrthoANI) value based on the whole-genome sequences was calculated using the EzBioCloud OAT tool (10). The OrthoANI values between the three *F. phosphoraccumulans* strains were ≥98.68%.

### Data availability.

The chromosome sequences, plasmid sequences, and reads of the three *F. phosphoraccumulans* strains were deposited in the GenBank/ENA/DDBJ database, and the details are shown in Table 1.

## References

[1] Watanabe K, Morohoshi S, Kunihiro T, Ishii Y, Takayasu L, Ogata Y, Shindo C, Suda W. 2020. *Fluviibacter phosphoraccumulans* gen. nov., sp. nov., a polyphosphate-accumulating bacterium of *Fluviibacteraceae* fam. nov., isolated from surface river water. Int J Syst Evol Microbiol 70:5551–5560. doi:10.1099/ijsem.0.004446. 32915122

[2] Watanabe K, Komatsu N, Kitamura T, Ishii Y, Park H-D, Miyata R, Noda N, Sekiguchi Y, Satou T, Atanabe M, Yamamura S, Imai A, Hayashi S. 2012. Ecological niche separation in the *Polynucleobacter* subclusters linked to quality of dissolved organic matter: a demonstration using a high sensitivity cultivation-based approach. Environ Microbiol 14:2511–2525. doi:10.1111/j.1462-2920.2012.02815.x. 22759205

[3] Watanabe K, Ishii Y, Komatsu N, Kitamura T, Watanabe M, Yamamura S, Imai A, Hayashi S. 2017. Growth rates and tolerance to low water temperatures of freshwater bacterioplankton strains: ecological insights from shallow hypereutrophic lakes in Japan. Hydrobiologia 792:67–81. doi:10.1007/s10750-016-3045-7.

[4] Watanabe K, Komatsu N, Ishii Y, Negishi M. 2009. Effective isolation of bacterioplankton genus *Polynucleobacter* from freshwater environments grown on photochemically degraded dissolved organic matter. FEMS Microbiol Ecol 67:57–68. doi:10.1111/j.1574-6941.2008.00606.x. 19049496

[5] Ogata Y, Suda W, Ikeyama N, Hattori M, Ohkuma M, Sakamoto M. 2019. Complete genome sequence of *Phascolarctobacterium faecium* JCM 30894, a succinate-utilizing bacterium isolated from human feces. Microbiol Resour Announc 8:e01487-18. doi:10.1128/MRA.01487-18. 30687834 PMC6346166

[6] Koren S, Walenz BP, Berlin K, Miller JR, Bergman NH, Phillippy AM. 2017. Canu: scalable and accurate long-read assembly via adaptive k-mer weighting and repeat separation. Genome Res 27:722–736. doi:10.1101/gr.215087.116. 28298431 PMC5411767

[7] Ogata Y, Sakamoto M, Ohkuma M, Hattori M, Suda W. 2020. Complete genome sequence of *Adlercreutzia* sp. strain 8CFCBH1, a potent producer of equol, isolated from healthy Japanese feces. Microbiol Resour Announc 9:e01240-20. doi:10.1128/MRA.01240-20. PMC7714862 33273007

[8] Wick RR, Judd LM, Gorrie CL, Holt KE. 2017. Unicycler: resolving bacterial genome assemblies from short and long sequencing reads. PLoS Comput Biol 13:e1005595. doi:10.1371/journal.pcbi.1005595. 28594827 PMC5481147

[9] Tanizawa Y, Fujisawa T, Nakamura Y. 2018. DFAST: a flexible prokaryotic genome annotation pipeline for faster genome publication. Bioinformatics 34:1037–1039. doi:10.1093/bioinformatics/btx713. 29106469 PMC5860143

[10] Lee I, Kim YO, Park SC, Chun J. 2016. OrthoANI: an improved algorithm and software for calculating average nucleotide identity. Int J Syst Evol Microbiol 66:1100–1103. doi:10.1099/ijsem.0.000760. 26585518

